# The Middlesex Training School

**Published:** 1920-10-09

**Authors:** 


### The Middlesex Training School.

Prize-giving was a very impressive ceremony this year. It took place on October 1, at La Scala Theatre, on the occasion of the opening of the autumn session of the Medical School. Her Royal Highness the Princess Alice, Duchess of Athlone, was present with the Duke of Athlone, Chairman of the hospital, and prizes were presented to the students and to successful nurses by Earl Beatty. Some pretty photographs were secured, and have been reproduced in the Press. Professor T. Swale Vincent delivered the opening address before a crowded audience. The medals, for which there is much competition, were designed as a memorial to the late Dr. Fardon, for so many years the main-spring of the hospital and a true friend to nurses. They are awarded to the nurses who obtain the highest marks on examination during their three years' training, added to marks for general efficiency and good conduct. The successful medallists were: Gold—Miss Helen Wisbey Wright; Silver—Miss Margaret Gardner; Bronze—Miss Edith Vidal Cox. We understand the fifty-six-hour week will shortly be introduced in the hospital for the day nurses. There are a few vacancies in the training-school for suitable candidates.

